# Genetic diagnosis of neurofibromatosis type 1: targeted next- generation sequencing with Multiple Ligation-Dependent Probe Amplification analysis

**DOI:** 10.1186/s12929-018-0474-9

**Published:** 2018-10-05

**Authors:** Yah-Huei Wu-Chou, Tzu-Chao Hung, Yin-Ting Lin, Hsing-Wen Cheng, Ju-Li Lin, Chih-Hung Lin, Chung-Chih Yu, Kuo-Ting Chen, Tu-Hsueh Yeh, Yu-Ray Chen

**Affiliations:** 1 Human Molecular Genetics Laboratory, Department of Medical Research, Chang Gung Memorial Hospital, No.5, Fushing Street, Kweishan, Taoyuan, Taiwan; 2 Division of Genetics and Endocrinology, Department of Pediatrics, Chang Gung University College of Medicine and Chang Gung Children’s and Memorial Hospital, No.5, Fushing Street, Kweishan, Taoyuan, Taiwan; 3 Department of Plastic & Reconstructive Surgery, Chang Gung Memorial Hospital, Kweishan, Taoyuan, Taiwan; 4 Neuroscience Research Center, Department of Neurology, Chang Gung Memorial Hospital, Taoyuan, Taiwan

**Keywords:** Neurofibromatosis type 1, RASopathies, Targeted NGS, MLPA, Genetic counseling

## Abstract

**Background:**

Neurofibromatosis type 1 (NF1) is a dominantly inherited tumor predisposition syndrome that targets the peripheral nervous system. It is caused by mutations of the *NF1* gene which serve as a negative regulator of the cellular Ras/MAPK (mitogen-activated protein kinases) signaling pathway. Owing to the complexity in some parts of clinical diagnoses and the need for better understanding of its molecular relationships, a genetic characterization of this disorder will be helpful in the clinical setting.

**Methods:**

In this study, we present a customized targeted gene panel of *NF1*/*KRAS*/*BRAF*/*p53* and *SPRED1* genes combined with Multiple Ligation-Dependent Probe Amplification analysis for the NF1 mutation screening in a cohort of patients clinically suspected as NF1.

**Results:**

In this study, we identified 73 *NF1* mutations and two *BRAF* novel variants from 100 NF1 patients who were suspected as having NF1. These genetic alterations are heterogeneous and distribute in a complicated way without clustering in either cysteine–serine-rich domain or within the GAP-related domain. We also detected fifteen multi-exon deletions within the *NF1* gene by MLPA Analysis.

**Conclusions:**

Our results suggested that a genetic screening using a NGS panel with high coverage of Ras–signaling components combined with Multiple Ligation-Dependent Probe Amplification analysis will enable differential diagnosis of patients with overlapping clinical features.

## Background

Neurofibromatosis type 1 (MIM# 162200) is a very common genetic disorder affecting approximately 1 in 3000–4000 individuals worldwide with the penetrance of the mutant gene being close to 100% by 5 years of age [1–4]. Clinically, it is presented with the occurrence of Café-au-lait macules, Lisch nodules, axillary freckling and multiple neurofibromas. Phenotypically, Neurofibromatosis type 1 (NF1) patients have a very heterogeneous condition. Discrete dermal neurofibromas occur in almost all adults with NF1, and the number usually increases with age. If whole-body magnetic resonance imaging (MRI) is used, plexiform neurofibromas are detectable in at least half of NF1 patients. Other complications include learning disabilities, mental retardation, optic gliomas, certain bone abnormalities, CNS tumors, and an increased risk for certain malignancies [5, 6].

NF1 is caused by mutations of the *NF1* gene which maps to chromosome 17q11.2. Many evidences have suggested *NF1* as a tumor suppressor gene as inactivation of both *NF1* alleles would reduce the control of cell proliferation and lead to tumorigenesis [7, 8]. The function of *NF1* gene product, neurofibromin, is to stimulate the GTPase activity of the RAS protein and serve as a negative regulator of the cellular Ras/MAPK (mitogen-activated protein kinases) signaling pathway [7, 9–11]. Up to date, more than 1000 pathogenic allelic variants have been identified in the *NF1* gene [The Human Gene Mutation Database (HGMD, Institute of Medical Genetics, Cardiff, http://www.hgmd.org/; Leiden Open Variation Database, LOVD: www.lovd.nl/NF1]. Most *NF1* mutations are single-base substitutions, insertions, or deletions. Other mutations are single- or multi-exon deletions or duplications and microdeletions encompassing NF1 and its neighboring genes [12–22].

NF1 is a member of RAS-related disorders, which usually show similar clinical features in cutaneous signs, cardiac defects, developmental disabilities and neurocognitive impairment [23–25]. Therefore, molecular diagnosis in NF1 should be of great value to confirm the diagnosis, particularly in the early childhood. However, the procedures for molecular diagnosis of NF1 are usually expensive, time-consuming, and labor-intensive [15–21, 26–28]. The development of next-generation sequencing (NGS) technologies which allows for rapid identification of disease-causing mutations and high-risk alleles has recently been introduced into NF1 diagnosis [29–34]. Owing to the complexity with some aspects of clinical diagnoses and the need for a better understanding of its molecular relationships, an extended genetic characterization of this disorder will be helpful in a clinical setting.

## Methods

### Patients and sample preparations

One hundred NF1 patients suspected as having NF1 by a clinical evaluation were recruited for this study. From each patient, 10 ml of whole blood samples were collected in EDTA-anticoagulant tube through the Linko Medical Center of the Chang Gung Memorial Hospital. Fifteen patients had a known family history of NF1. Ethical approval for this study was obtained by the institutional review board (102-0226A3) at Chang Gung Memorial Hospital. All participants provided written informed consent. Genomic DNA of each patient was then prepared using the PUREGENE DNA purification kit from GENTRA using standard protein precipitation procedures. The quality of the DNA was estimated using the Nano-Drop spectrophotometer (Thermo Fisher Scientific, Waltham, MA, USA).

### Candidate gene-targeted sequencing

A panel of five NF1-related genes including *NF1* (NM_000267, 17q11.2), *SPRED1* (NM_152594, 15q14), *KRAS* (NM_004985, 12p12.1), *BRAF* (NM_004333, 7q34), and *p53* (NM_000546, 17p13.1) was initially created designed to capture, amplify, and sequence specific regions (including exons and splice junctions) of the genome for human cancer screening. The total length was 32.3 kb encompassing 296 amplicons, and the coverage was 507×. Adapter sequences were clonally amplified by emulsion PCR on the high-density array of micro-machined wells. In this study, we took the advantage of this gene panel for the germline mutation analysis of NF1 using the Ion Personal Genome Machine® (PGM™) Sequencer (Life technology).

### Sample library preparation

A total of 100 indexed rapid prepared Ion AmpliSeq DNA libraries, starting from 100 ng of gDNA per sample, were prepared according to the manufacturer’s instructions. Template preparation and emulsion PCR and Ion Sphere Particles (ISP) enrichment were performed according to the manufacturer’s instructions. Following the purification and size selection using AMPure beads (Beckman Coulter, Brea, CA, USA), the size distribution of the DNA fragments was analyzed on the Agilent Bioanalyzer using High-Sensitivity DNA chip (Agilent Technologies Inc., Santa Clara, CA) and the quality checking of ion sphere particles for the prepared library was performed using Qubit 2.0 Fluorometer (Life Technologies). Enriched ISPs were prepared for sequencing using the Ion PGM 200 Sequencing Kit v2.0 and were loaded on an Ion 316 chip v2 or Ion 318 chip v2.

### Data analysis

We used IT platform-specific pipeline software Torrent Suite, version 4.2, with the plug-in “variant caller” program (Life Technologies) to perform reference genome alignment, base calling, and filtering of poor signal reads. The Integrative Genome Viewer (IGV) (http://software.broadinstitute.org/software/igv/) was used for visualizing the status of each read alignment. The selected variants were classified as deleterious mutation by mutation type if they were identified as nonsynonymous, frameshift, or stopgain at the exonic region. ACMG Standards and Guidelines for the interpretation of sequence variants were followed in this study [35]. In an appropriate reference population, the pathogenic variant should have a frequency of much less than 1%. We removed all the common variants (Minor Allele Frequency, MAF > 1%) reported in the following public databases: 1000 Genomes Project (http://www.1000genomes.org/), dbSNP database and ClinVar database (https://www.ncbi.nlm.nih.gov/snp/; https://www.ncbi.nlm.nih.gov/clinvar/). Variants with amino acid changes were further examined for whether the changes were potentially damaging alterations using Sorting Tolerant From Intolerant (SIFT) and Polymorphism Phenotyping v2 (PolyPhen2) softwares, which can predict the possible impact of an amino acid substitution on the structure and function of a protein. The nomenclature of novel variants followed the rules of the Human Genome Variation Society (http://www.hgvs.org/mutnomen/). The genetic variants in the Human Gene Mutation Database (HGMD, Institute of Medical Genetics, Cardiff, http://www.hgmd.org/) and Leiden Open Variation Database (LOVD: www.lovd.nl/NF1) were also considered as references.

### PCR amplification and sanger sequencing verification

We performed Sanger validation for all putatively pathogenic SNVs and indels variants on each detected patient (and their family members, if available) by PCR amplification and sequenced with Applied Biosystems 3730 Genetic Analyzer. PCR amplification was performed under standard conditions with 30 PCR cycles and 55°–60 °C annealing. PCR products were sequenced using the Big Dye Terminator cycle sequencing kit (Life Technologies) according to the manufacturer’s cycling conditions and analyzed on an Applied Biosystems 3730xl Automated Sequencer Genetic Analyzer (Life Technologies). Sequence alignments and analysis were further performed using the Autoassembler computer program (Life Technologies).

### Multiplex ligation-dependent probe amplification (MLPA) analysis

We used SALSA P081/P082 NF1 MLPA kit (MRC Holland, Amsterdam, The Netherlands) to confirm and identify single and multiple exon deletions/duplications according to the manufacturer’s protocol. Each samples containing 100 ng of genomic DNA was used for overnight hybridization with the probemixes. After ligation and amplification were performed with FAM-labeled primers, the PCR products were analyzed on a Genetic Analyzer 3730 capillary electrophoresis system and interpreted using Genotyper version 2.0 (Applied Biosystems, CA, USA). In this study, we used the Coffalyser program (version 3.5) for peak area normalization and gene dosage calculation.

## Results

### Genetic alterations identified from a targeted NGS gene panel screening

A total of 100 individuals from 95 families who were clinically suspected as carrying NF1 were referred for this genetic testing. A brief summary of the clinical data collected for each patient is given in Table 1. Fifteen patients (15%) had a family history of NF1 in this cohort. Café-au-lait spots and Lisch nodules in the iris were observed in 93 and 19 patients, respectively. Cutaneous neurofibromas, plexiform neurofibromas and malignant peripheral nerve sheath tumors were identified in 32, 13, and 2, patients, respectively. Five patients had optic gliomas and two patients had brain tumors. Among these individuals, we have identified seventy-three *NF1* mutations (Table 2) and two *BRAF* novel variants from a targeted NGS gene panel of *NF1*/*KRAS*/*BRAF*/*p53* and *SPRED1* analyses. *SPRED1* genetic mutations were not detected in this study. Variants with amino acid changes were further examined to check if the changes were potentially damaging alterations using Sorting Intolerant from Tolerant (SIFT) algorithm and Polymorphism Phenotyping v2 (PolyPhen2) software, which can predict the possible impact of an amino acid substitution on the structure and function of a protein.

**Table 1:** Clinical features of 100 Taiwanese NF1 patients

Clinical features	Patients^a^ (%)
Café-au-lait spots	93 (93%)
Lisch nodules in the Iris	19 (19%)
Cutaneous neurofibroma	32 (32%)
Plexiform neurofibroma	13 (13%)
Malignant peripheral nerve sheath tumor	2 (2%)
Optic glioma	5 (5%)
Brain tumor	2 (2%)
Scoliosis	10 (10%)
Heart defects	8 (8%)
Learning disability	4 (4%)
Craniofacial disability	9 (9%)
Family history	15 (15%)

^a^11 patients are under 12 years old; male: female = 53:47

**Table 2:** *NF1* Mutational profile of the 100 NF1 blood samples tested in NGS study

Patient	Coding^a^	Amino Acid Change	Variant Effect	NM_000267.3	SIFT	Polyphen2
Wu p001	**c.492_495 del AACT/Het**	p.Val166fs	Frameshift Deletion	Exon 5		
Wu p002	**c.5844C > A**	p.Tyr1948Ter	nonsense	Exon 40		
Wu p003	c.1466A > G, **c.1400C > T, c.1448A > G, c.1513A > G**	p.Tyr489Cys	missense	Exon 13	Tolerated	Benign
Wu p004	**c.6855C > A**	p.Tyr2285Ter	nonsense	Exon 46	Tolerated	
Wu p006	**c.2982_2982delT**	p.Leu995fs	Frameshift Deletion	Exon 22		
Wu p007	c.1105C > T	p.Gln369Ter	nonsense	Exon 10	Tolerated	
Wu p009	**c.7862_7862delC**	p.Thr2621fs	Frameshift Deletion	Exon 54		
Wu p010	**c.5902C > T**	p.Arg1968Ter	nonsense	Exon 40	Tolerated	
Wup011	**c.7152_7157del TAACTT**	p.2384_2386del	Deletion	Exon 49		
Wu p014	c.3113 + 1 G > A	.	splicing	Exon 23		
Wu p015	**c.4700C > G**	p.Ser1567Ter	nonsense	Exon 36	Tolerated	
Wu p017	**c.487G > T**	p.Glu163Ter	nonsense	Exon 5		
Wu p018	**c.6970C > T**	p.Gln2324Ter	nonsense	Exon 47	Damaging	
	**c.8386A > C**	p.Lys2796Gln	missense	Exon 58	Damaging	Possibly damaging
	**c.8520 + 125 del C (Intron)**		Frameshift Deletion	Exon 58		
Wu p019	**c.575G > A**	p.Arg192Gln	missense	Exon 5	Tolerated	Benign
	**c.1422_1422delC**	p.Lys476fs	Frameshift Deletion	Exon 13		
Wu p021	**c.1080_1083delAAGT**	p.Lys362fs	Frameshift Deletion	Exon 10		
Wu p022	c.1062G > C	p.Lys354Asn	missense	Exon 9	Tolerated	Possibly damaging
Wu p023	c.1062G > C	p.Lys354Asn	missense	Exon 9	Tolerated	Possibly damaging
Wu p024	c.1658A > C	p.His553Pro	missense	Exon 15	D	Possibly damaging
Wu p025	**c.4316 T > A**	p.Leu1439Ter	nonsense	Exon 32	Tolerated	
Wu p027	**c.1754_1757delTAAC**	p.Thr586fs	Frameshift Deletion	Exon 16		
Wu p030	**c.5665G > T**	p.Glu1889Ter	nonsense	Exon 39	Tolerated	
Wu p032	c.2266C > T	p.Gln756Ter	nonsense	Exon 19	Damaging	
Wu p033	**c.7348C > T**	p.Arg2450Ter	nonsense	Exon 50	Tolerated	
Wu p034	c.910C > T	p.Arg304Ter	nonsense	Exon 9	Tolerated	
Wu p035	**c.5580_5581insA**	p.Asn1861fs	Frameshift Insertion	Exon 38		
Wu p038	c.1246C > T	p.Arg416Ter	nonsense	Exon 11	Tolerated	
Wu p039	**c.492_495 del AACT/He**	p.Val166fs	Frameshift Deletion	Exon 5		
Wu p041	c.910C > T	p.Arg304Ter	nonsense	Exon 9	Tolerated	
Wu p043	**c. 3796 G > T**	p.Glu1266Ter	nonsense	Exon28	Tolerated	
Wu p044	**c.86_87delAC**	p.29_29del	frameshift deletion	Exon2		
Wu p045	**c.6618_6618 delA**	p.Thr2206fs	frameshift deletion	Exon43		
Wu p047	**c. 6818 A > C**	p.Lys2273 Thr	missense	Exon46	Tolerated	Possibly damaging
Wu p048	c. 910 C > T	p.Arg304Ter	nonsense	Exon9	Tolerated	
Wu p050	**c.2212dupT**	p.Phe738fs	frameshift insertion	Exon18		
Wu p051	**c. 5170 C > T**	p.Gln1724Ter	nonsense	Exon37	Tolerated	
Wu p052	**c. 1224 T > A**	p.Tyr408Ter	nonsense	Exon11	Tolerated	
Wu p053	**c.7266_7267del AC**	p.2422_2423del	frameshift deletion	Exon49		
Wu p054	c. 574 C > T	p.Arg192Ter	nonsense	Exon5	Tolerated	
Wu p055	c. 574 C > T	p.Arg192Ter	nonsense	Exon5	Tolerated	
Wu p058	**c. 3040 A > T**	p.K1014Ter	nonsense	Exon23		
Wu p059	c.288 + 1G > T	.	splicing	Exon3		
Wu p060	**c.4509dupT**	p.Asn1503fs	frameshift insertion	Exon34		
Wu p064	c. 479 G > T	p.Arg160Met	missense	Exon4	Damaging	Possibly damaging
Wu p066	**c.1592delA**	p.Gln531fs	frameshift deletion	Exon14		
Wu p067	**c.8070dupC**	p.Tyr2690fs	frameshift insertion	Exon56		
Wu p068	c.288 + 1G > T	.	splicing	Exon3		
Wu p070	**c.4990_4992AAA (GTT)**	.	nonframeshift substitution	Exon37		
Wu p071	c. 3826 C > T	p.Arg1276Ter	nonsense	Exon28	Tolerated	
Wu p073	**c.2340_2346delACATGCA**	p.780_782del	frameshift deletion	Exon20		
Wu p074	**c. 4107 C > A**	p.Tyr1369Ter	nonsense	Exon30	Tolerated	
Wu p075	**c. 5651 T > G**	p.Phe1884Cys	missense	Exon39	Damaging	Damaging
Wu p076	**c. 3888 T > G**	p.Tyr1296Ter	nonsense	Exon29	Tolerated	
Wu p077	**c. 3484 A > G**	p.Met1162Val	missense	Exon26	Tolerated	Benign
Wu p077	**c. 7189 G > A**	p.Gly2397Arg	missense	Exon49	Damaging	Damaging
Wu p080	c. 1933 A > G	p.Met645Val	missense	Exon17	Tolerated	Benign
Wu p081	**c.1754_1757del**	p.Leu585fs	frameshift deletion	Exon16		
Wu p083	**c.2953dupC**	p.Gly984fs	frameshift insertion	Exon22		
Wu p086	**c.6855C > A**	p.Tyr2285Ter	nonsense	Exon46	Tolerated	
Wu p087	**c. 4940 A > C**	p.His1647Pro	missense	Exon37	Tolerated	Damaging
Wu p088	**c.1754_1757del**	p.Leu585fs	frameshift deletion	Exon16		
Wu p089	c. 1466 A > G	p.Tyr489Cys	missense	Exon13	Tolerated	Damaging
Wu p090	c. 376 G > T	p.Glu126Ter	nonsense	Exon4	Damaging	
Wu p092	c. 3827 G > A	p.Arg1276Gln	missense	Exon28		Damaging
Wu p094	**c. 3796 G > T**	p.Glu1266Ter	nonsense	Exon28	Tolerated	Damaging
Wu p095	**c.1693dupG**	p.Asp564fs	frameshift insertion	Exon15		
Wu p098	**c.1754_1757del**	p.Leu585fs	frameshift deletion	Exon16		
Wu p100	c. 1318 C > T	p.Arg440Ter	nonsense	Exon12	Tolerated	

^a^bold lettering indicated as novel variants

**Fig. 1:**
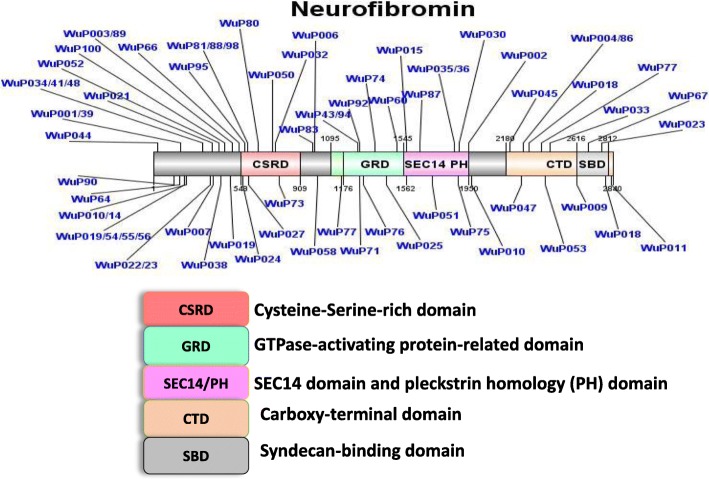
Details of the 73 *NF1* genetic variations identified by NGS targeted gene sequencing. The position of genetic variations detected in the *NF1* gene from each patient is shown and their relationship to a possible defect of NF1 gene was also included. Known functional domains of Neurofibromin: CSRD > cysteine–serine-rich domain; GRD > GTPase-activating protein-related domain; SEC14/PH > SEC14 domain and pleckstrin homology (PH) domain; CTD > Carboxy-terminal domain; SBD > Syndecan-binding domain

**Fig. 2:**
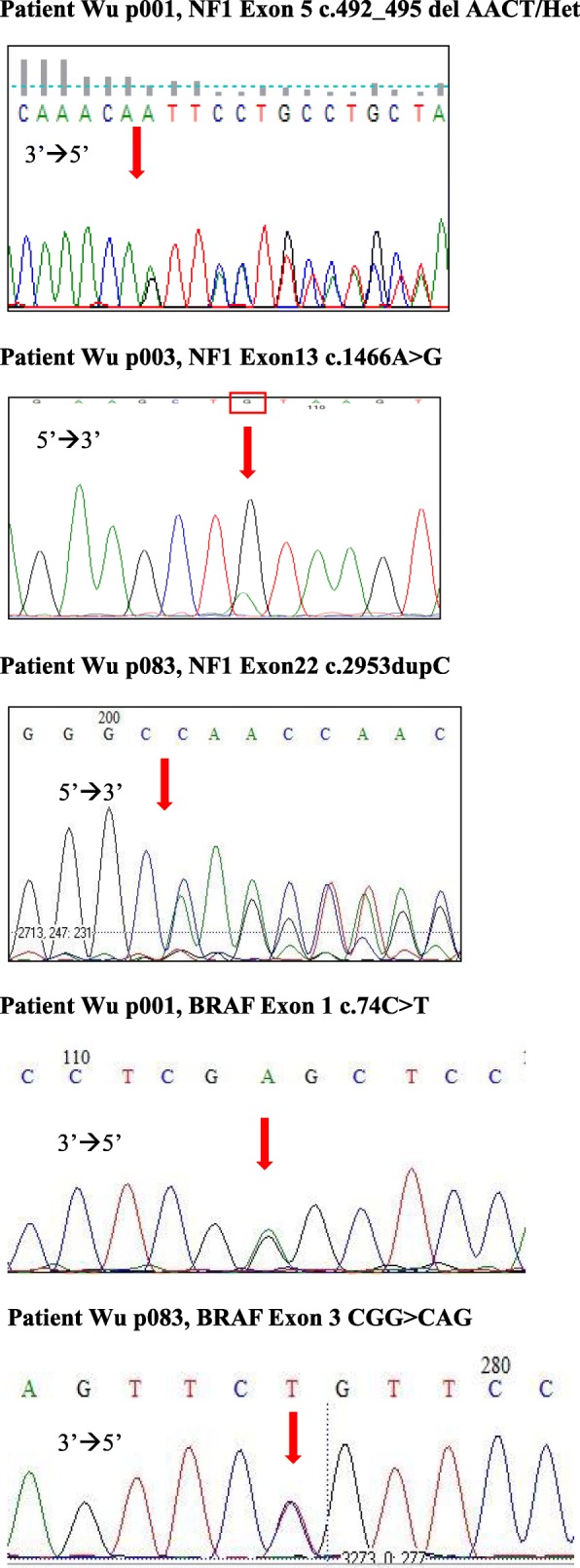
Some represented results of Sanger sequencing at the mutation site with blood sample

Genetic alterations in the *NF1* gene were detected as frameshift, nonsense, splice, missense mutations, and frame deletions or duplications from the first NGS panel screening (Fig. 1). These variants distributed along the *NF1* gene without any clustering hotspot domain. Intragenic *NF1* point mutations were found in 46 patients, 28 nonsense and 18 missense mutations. Small insertions and/or deletions were identified in 24 patients and most of them with frameshift consequences. Splice alterations were detected only in three patients. Four patients (Wu p003, Wu p018, Wu p019, and Wu p077) possessed more than one *NF1* variant. Two patients with *BRAF* variants (c.74C > T in Exon1: p.Pro25Leu; c.G316A in Exon 3: p.Gly106Arg) were identified from this NGS screening. Both these patients also carried *NF1* mutations (Fig. 2). On comparing with the Human Gene Mutation Database (HGMD, Institute of Medical Genetics, Cardiff, http://www.hgmd.org/), and Leiden Open Variation Database (LOVD: www.lovd.nl/NF1), we found that 48 variants of *NF1* gene and two of *BRAF* gene are supposed to be novel (presented in bold in Table 2 and Table 3). All these novel mutations in this study were tested in 100 normal alleles.

**Table 3:** *NF1* multi-exon deletions or duplications

NAME	MLPA	Clinical features	Tumor type
Wu p008	3’ UTR del/He	café-au-lait spot	Multiple cutaneous tumor
Wu p013	Exon 10 ~ 58 del/He	café-au-lait spot	whole body
Wu p016	Exon 1 ~ 58 del/He	café-au-lait spot, skin nodules	Two nodules of tumor involving the dermis and composed of spindle cells with wavy elongated nuclei
Wu p020	Exon 28~ 39 del/He	café-au-lait spot	Neurofibroma
Wu p029	Exon 4C~ 6 (no Exon 5)	café-au-lait spot, skin nodules	Right facial plexiform neurofibroma
Wu p031	Exon 1B~ 49	café-au-lait spot, skin nodules	multiple nodules over face and bilateral forearms
Wu p037	Exon1~ 58 del/He	café-au-lait spot/List Nodules in the Iris	multiple nodules over face
Wu p061	Exon37~ 51 del/He	café-au-lait spot	NF1 with optic nerve glioma
Wu p062	Exon2~ 8 del/He	café-au-lait spot/List Nodules in the Iris	right thigh subcutaneous layer soft tissue nodule
Wu p065	Exon 28–29 del/He	café-au-lait spot	lower limb plexiform NF
Wu p069	Exon1~ 58 del/He	café-au-lait spot/List Nodules in the Iris	Neurofibroma over back
Wu p082	Exon2~ 5 del/He	café-au-lait spot	plexiform neurofibroma over buttock
Wu p085	Exon2~ 5 del/He	café-au-lait spot	left optic nerve glioma & liposarcoma
Wu p093	Exon 1B ~ 4B	skin nodules/List Nodules in the Iris	Plexiform Neurofibroma
Wu p099	Exon 4C~ 6 (no Exon 5)	café-au-lait spot, skin nodules	skin and soft tissue on right face, plexiform neurofibroma

### Spectrum of *NF1* mutations identified by MLPA analysis

**Fig. 3:**
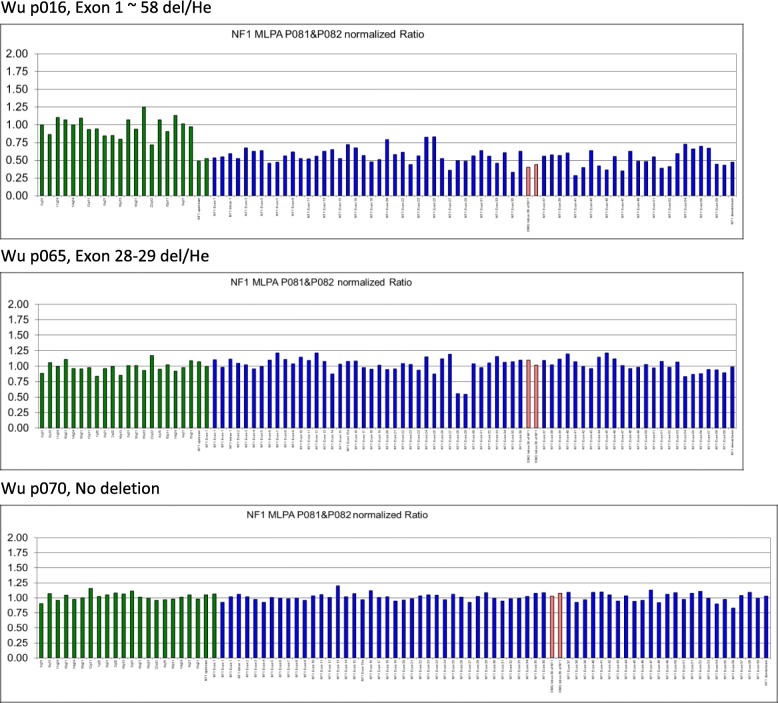
Examples of multi-exon deletions detected by multiplex ligation-dependent probe amplification. In this study, we used the Coffalyser program (version 3.5) for peak area normalization and gene dosage calculation. Two copies of the genome have a relative peak area value of approximately 1.0. A reduction in the peak area value to < 0.7 indicates the occurrence of a deletion

For patients who showed no detected mutations by our NGS panel screening, we then analyzed possible exons deletion/duplication within the *NF1* gene using multiplex ligation-dependent probe amplification (MLPA) approach. Whole *NF1* gene deletions were found in three patients and fifteen multi-exon deletions within the *NF1* gene were obtained in this cohort of NF1 patients. Most of these exon deletions were only seen once in this study (Fig. 3, Table 3).

### Clinical features of NF1 patients with concurrence of *NF1-BRAF* mutations

Patient (Wu p001) was diagnosed as NF1 at the age of seven years by the presence of left craniofacial plexiform neurofibromas, infiltrative at the left temporal scalp, nodular subcutaneous tissue of the cheek, masticator space and probably the parotid space. He had multiple café-au-lait spots but no Lisch nodules. His brain magnetic resonance image (MRI) showed multiple unknown bright objects at pons, bilateral cerebellar hemisphere, globus pallidus and right thalamus. His left temporal and zygomatic bone showed progressive enlargement. His father (Wu p039) was the first patient with NF1 in this family and was diagnosed as having NF1 because of the presence of Lisch nodules, skeletal dysplasia, hundreds of café-au-lait spots and cutaneous nodular neurofibromas all over the body. This father and son are both intellectually normal. They both share the same genetic alterations on *NF1* (c.492_495 del AACT/p.Val166fs) and *BRAF* (c.74C > T/p.Pro25Leu) gene (Fig.2). Another patient (Wu p083) was diagnosed as having NF1 at age of five years. He had multiple café-au-lait spots and Lisch nodules with soft tissue mass over the right back. He also had unspecified heart anomaly and T-spine scoliosis. His brain MRI showed white matter hyperintensity suggesting spongiform change at the left globus pallidus, dorsal pons, and bilateral cerebellar hemisphere (dentate nuclei) but no definite evidence of optic gliomas. He was detected as having *NF1* (c.2953dupC/p.Gly984fs) and *BRAF* (c.316 G > A/p.Gly106Arg) genetic variants in the first NGS screening (Fig. 2, Table 2). These three patients presented no data for their definite atrial septal defect, ventricular septal defect and patent ductus arteriosus. In addition, none of these patients show the typical features of NF1–Noonan syndrome, Noonan syndrome or CFC syndrome.

## Discussion

We here assessed a DNA-based approach combining targeted gene panel screening with MLPA analysis in a cohort of clinically suspected NF1 patients. On targeted gene panel screening, we identified 73 *NF1* mutations and two *BRAF* variants (c.74C > T: p.Pro25Leu; c.316 G > A: p.Gly106Arg) in a total of 100 NF1 patients from 95 families diagnosed as having NF1 on the basis of the clinical criteria. These mutations are heterogeneous and distribute without clustering in either cysteine–serine-rich domain or within the GAP-related domain. For patients in whom mutations were not detected by our NGS panel screening, we detected fifteen multi-exon deletions within the *NF1* gene by Multiplex Ligation-Dependent Probe Amplification (MLPA) analysis (~ 15% of detected *NF1* alterations). A multi-step mutation detection protocol has been used for over 95% of pathogenic *NF1* mutations in different laboratories [15–21, 26–28]. The *NF1* mutations were detected in our study was in 92.6% (88/95) of the subjects when five patients who did not completely met the clinical diagnostic criteria were excluded. Our analysis and this study may have missed the genetic variants residing in the promotor and intronic untranscribed non-coding regions or those involved in large genomic rearrangements or epigenetic mechanisms. We anticipate that whole-genome analysis may provide further insights for the information related to this issue.

NF1 is a progressive disorder complicated by the variability of disease expression. Beyond the primary concern of cutaneous/dermal neurofibromas, pigmented lesions, and the occasional limb abnormalities, the majority of NF1 patients do not fulfill the NIH criteria. Only ~ 30% of NF1 patients develop clinically detectable plexiform neurofibromas, and many features of NF1 only display café-au-lait spots and mild symptoms or no major disease complications in their early life [5, 36, 37]. Although neurofibromatosis type 1 is the most common syndrome seen in children with multiple café-au-lait spots, other syndromes associated with one or more café-au-lait spots include McCune-Albright syndrome, Legius syndrome, Noonan syndrome and other neuro-cardio-facio-cutaneous syndromes [38]. It also shares some features including reduced growth, facial dysmorphia, cardiac defects, skeletal and ectodermal anomalies, variable cognitive deficits, and susceptibility to certain malignancies with a group of clinically distinct developmental disorders [23–25]. Neurofibromatosis type I, Noonan syndrome, LEOPARD syndrome, and cardiofaciocutaneous syndromes were usually grouped as “neuro-cardio-facio-cutaneous” (NCFC) syndromes but are now called as “RaSopathies”. All these disorders involve a common Ras–Raf–signaling pathway [39–41]. To our knowledge, germline *KRAS* mutations occasionally occur in Noonan (2–4%) [42–46] and CFC syndromes (< 2%) [43–45, 47, 49]. Germline *BRAF* mutations can cause CFC syndrome (approximately 50–75%) [44, 47–50], Noonan syndrome [47, 50], and LEOPARD syndrome type 3 (< 2%) [50, 51]. However, these individuals usually are not associated with neurofibromas (Table 4).

**Table 4:** *BRAF* mutations in patients with RASopathies

Patient	Germline mutation	Clinical Phenotypes	Tumor type
Wu p001 (this study)	*NF1* Exon 5, **c.492_495 del AACT/p.Val166fs**	Café-au-lait spots, Cutaneous neurofibroma, left zygoms progressive enlargement	plexiform neurofibroma
	*BRAF* Exon 1, **c.74C > T/p.Pro25Leu**		
Wu p083 (this study)	*NF1* Exon22, **c.2953dupC/p.Gly984fs**	Café-au-lait spots, unspecified cardiac anomaly, Lisch Nodules in the Iris, T-spine scoliosis	paraspinal plexiform neurofibroma
	*BRAF* Exon 3, c. 316 **G > A/p.Gly106Arg**		
Noonan syndrome (NS)	*BRAF* (T241 M; T241R; W531C; L597 V)	Short stature, dysmorphic facial features, mild-to-moderate cognitive deficits, skeletal anomalies, and hypotonia	
Cardio-facio-cutaneous syndrome (CFCS)	*BRAF* (L245F; A246P; T241P; Q257R; G469E; etc)	Dysmorphic facies, cardiac defects, and skin and skeletal anomalies	
Leopard syndrome Type 3	*BRAF* (T241P; L245F)	Craniofacial anomalies, short and webbed neck, cardiac conduction defects, Multiple pigmented skin lesions and showed growth retardation, delayed puberty, and delayed bone age.	undetected

*bold lettering indicated as novel variants

Phenotypic variation could result from different expression patterns of mutated genes, as well as from different mechanisms that disturb RAS signaling through specific interactions with effector and regulatory proteins for different mutants. Variability could also result from the feedback mechanisms that can affect upstream molecules (like RAS) but not downstream molecules [40]. Therefore, a NGS panel with high coverage of Ras–signaling components should be very useful in clinical diagnosis. However, we cannot yet explain how the concurrence of *NF1* and *BRAF* variants contributes to NF1 in these patients.

## Conclusion

Differential diagnosis of NF1-like patients is still challenging owing to its clinical complexity. A genetic screening using a NGS panel in high coverage of Ras–signaling components combined with Multiple Ligation-Dependent Probe Amplification analysis should enable us to get the molecular control of these clinically overlapping disorders. We believe that the availability of whole-genome analysis will provide an opportunity for the genetic diagnosis of NF1 and will bring more insights for the development of NF1.

## Abbreviations

CSRD: Cysteine–serine-rich domain
CTD: Carboxy-terminal domain
GAP-related domain: GTPase activating protein-related domain
GRD: GTPase-activating protein-related domain
HGMD: Human Gene Mutation Database
IGV: Integrative Genome Viewer
indels variants: insertions/deletions variants
ISP: Ion Sphere Particles
LOVD: Leiden Open Variation Database
MAF: Minor Allele Frequency
MLPA: Multiple Ligation-Dependent Probe Amplification
MRI: Magnetic Resonance Image
NCFC syndromes: Neuro-Cardio-Facio-Cutaneous syndromes
NF1: Neurofibromatosis type 1
NGS: Next-Generation Sequencing
PCR: Polymerase Chain Reaction
PH: pleckstrin homology domain
PolyPhen2: Polymorphism Phenotyping v2
Ras/MAPK signaling pathway: Ras/Mitogen-Activated Protein Kinases signaling pathway
SBD: Syndecan-binding domain
SEC14/PH: SEC14 domain and Pleckstrin Homology domain
SIFT: Sorting Intolerant from Tolerant
SNVs: Single Nucleotide Variants
